# Severe thermal and major traumatic injury results in elevated plasma concentrations of total heme that are associated with poor clinical outcomes and systemic immune suppression

**DOI:** 10.3389/fimmu.2024.1416820

**Published:** 2024-06-14

**Authors:** Sebastian Tullie, Thomas Nicholson, Jonathan R. B. Bishop, Kirsty C. McGee, Ali Asiri, Jack Sullivan, Yung-Yi Chen, Amanda V. Sardeli, Antonio Belli, Paul Harrison, Naiem S. Moiemen, Janet M. Lord, Jon Hazeldine

**Affiliations:** ^1^ Institute of Inflammation and Ageing, University of Birmingham, Birmingham, United Kingdom; ^2^ Institute of Applied Health Research, University of Birmingham, Birmingham, United Kingdom; ^3^ National Institute for Health Research Surgical Reconstruction and Microbiology Research Centre, Queen Elizabeth Hospital Birmingham, Birmingham, United Kingdom; ^4^ University Hospital Birmingham National Health Service (NHS) Foundation Trust, Queen Elizabeth Hospital Birmingham, Birmingham, United Kingdom; ^5^ The Scar Free Foundation Centre for Conflict Wound Research, Queen Elizabeth Hospital Birmingham, Birmingham, United Kingdom; ^6^ Medical Research Council (MRC)-Versus Arthritis Centre for Musculoskeletal Ageing Research, University of Birmingham, Birmingham, United Kingdom

**Keywords:** burns, critical care, heme, immune suppression, trauma

## Abstract

**Background:**

Traumatic and thermal injuries result in a state of systemic immune suppression, yet the mechanisms that underlie its development are poorly understood. Released from injured muscle and lysed red blood cells, heme is a damage associated molecular pattern with potent immune modulatory properties. Here, we measured plasma concentrations of total heme in over 200 traumatic and thermally-injured patients in order to examine its relationship with clinical outcomes and post-injury immune suppression.

**Methods:**

Blood samples were collected from 98 burns (≥15% total body surface area) and 147 traumatically-injured (injury severity score ≥8) patients across the ultra-early (≤1 hour) and acute (4–72 hours) post-injury settings. Pro-inflammatory cytokine production by lipopolysaccharide (LPS) challenged whole blood leukocytes was studied, and plasma concentrations of total heme, and its scavengers haptoglobin, hemopexin and albumin measured, alongside the expression of heme-oxygenase-1 (HO-1) in peripheral blood mononuclear cells (PBMCs). LPS-induced tumour necrosis factor-alpha (TNF-α) production by THP-1 cells and monocytes following *in vitro* heme treatment was also examined.

**Results:**

Burns and traumatic injury resulted in significantly elevated plasma concentrations of heme, which coincided with reduced levels of hemopexin and albumin, and correlated positively with circulating levels of pro and anti-inflammatory cytokines. PBMCs isolated from trauma patients 4–12 and 48–72 hours post-injury exhibited increased HO-1 gene expression. Non-survivors of burn injury and patients who developed sepsis, presented on day 1 with significantly elevated heme levels, with a difference of 6.5 µM in heme concentrations corresponding to a relative 52% increase in the odds of post-burn mortality. On day 1 post-burn, heme levels were negatively associated with *ex vivo* LPS-induced TNF-α and interleukin-6 production by whole blood leukocytes. THP-1 cells and monocytes pre-treated with heme exhibited significantly reduced TNF-α production following LPS stimulation. This impairment was associated with decreased gene transcription, reduced activation of extracellular signal-regulated kinase 1/2 and an impaired glycolytic response.

**Conclusions:**

Major injury results in elevated plasma concentrations of total heme that may contribute to the development of endotoxin tolerance and increase the risk of poor clinical outcomes. Restoration of the heme scavenging system could be a therapeutic approach by which to improve immune function post-injury.

## Introduction

Two opposing clinical syndromes characterise the immune and inflammatory response to major traumatic and thermal injury: a pro-inflammatory systemic inflammatory response syndrome (SIRS) and a counteracting compensatory anti-inflammatory response syndrome (CARS). Evident within minutes of trauma, and persisting into the acute injury phase (1–4), a defining feature of the CARS response is reduced *ex vivo* production of pro-inflammatory cytokines by lipopolysaccharide (LPS) challenged monocytes (5). Associated with the development of nosocomial infections (NI) and sepsis (6), this post-injury induction of endotoxin tolerance is of clinical significance, yet the mechanisms underlying its development are poorly understood.

Offering a potential explanation for the state of systemic immune suppression that develops post-trauma is the concept of “damage associated molecular pattern (DAMP)-induced immune tolerance”. Culminating in impaired anti-microbial responses to secondary stimulation, this theory proposes that following their release from injured tissues, DAMPs, a heterogeneous collection of nuclear, cytosolic and mitochondrial-derived proteins, lipids and DNA, induce functional tolerance in circulating immune cells through binding to pathogen recognition receptors (PRR) (7, 8). Suggesting a role for endogenous ligands of toll-like receptor 4 (TLR4) in mediating post-injury endotoxin tolerance, pre-conditioning monocytes isolated from healthy volunteers with DAMPs detected by this PRR, such as high mobility group box-1 (HMGB-1), heat shock protein-70 (HSP-70) and calprotectin, has been shown to significantly reduce their production of tumour necrosis factor-alpha (TNF-α) upon subsequent LPS stimulation (9–12).

Involved in oxygen transport and energy production, heme is an iron containing intracellular porphyrin that serves as the prosthetic group for such haemoproteins as myoglobin, haemoglobin and cytochrome P450. However, in times of cellular and tissue damage, heme is released from haemoproteins into the extracellular environment, where it functions as a potent immunomodulatory molecule. In conditions associated with significant haemolysis or rhabdomyolysis, free heme, a ligand of TLR4 (13), has been proposed to promote systemic inflammation by triggering the generation of pro-inflammatory cytokines, reactive oxygen species (ROS) and extracellular traps by neutrophils and macrophages (13–16). Recently, immune tolerising properties have also been assigned to heme, with murine models of trauma haemorrhage and liver crush injury demonstrating that elevated circulating concentrations of this DAMP were associated with an increased susceptibility to, and severity of, pulmonary infections (17, 18). Attributed to impaired innate immune responses, heme was shown to suppress the phagocytic activity of alveolar macrophages (18) and promote the downregulation of TLR2 and TLR4 from the neutrophil surface (17). Whether heme induced a state of systemic endotoxin tolerance post-injury was not investigated.

At present, our understanding of how trauma impacts upon circulating heme levels is based on the results of a single study. Performed alongside a murine model of liver crush injury, in which an immediate (<30 minutes) elevation in plasma heme levels was detected, Lee et al. analysed post-hospital admission plasma samples from six trauma patients and reported a significant post-injury increase in heme concentrations (17). Notably, despite profound tissue injury and haemolysis (19), no study, to our knowledge, has measured circulating heme levels in thermally-injured patients. Rather, studies have focused upon burn-induced changes in the heme scavenging system (20–22). Comprised of the plasma proteins hemopexin, haptoglobin and albumin, and the inducible enzyme heme oxygenase-1 (HO-1), this scavenging system is responsible for the neutralisation, degradation and removal of free heme (23, 24). A prospective based study of five severe burns patients detected significantly lower concentrations of hemopexin in plasma samples obtained at days 1–5 post-injury (20), whilst an analysis of skin biopsies obtained from eleven patients revealed a post-burn increase in HO-1 expression (22). Whether traumatic injury leads to alterations in the scavenging of free heme is currently unclear, with results of human and murine-based studies reporting either no difference or a significant increase in plasma hemopexin levels post-injury (17). Severe trauma has however been shown to increase HO-1 expression in circulating leukocytes, with this post-injury induction preceding the diagnosis of sepsis (25, 26).

Investigating to what extent free heme contributes to immune dysfunction after traumatic injury was recently identified as a high priority research topic in a scoping review that discussed the role of hemolysis in the pathophysiology of trauma (27). It has been suggested that, by reducing anti-microbial immune responses, excessive heme release combined with a post-injury dysregulation in heme scavenging may contribute to the increased susceptibility of critically ill patients to NI and sepsis (25). To test this hypothesis, we have performed, for the first time, a comprehensive assessment of the impact of severe injury on heme biology. Combined with *ex vivo* analyses of LPS-induced cytokine production by whole blood leukocytes, and an assessment of HO-1 expression in peripheral blood mononuclear cells (PBMCs) of major trauma patients, we have measured the concentrations of total heme, hemopexin, haptoglobin and albumin in plasma samples obtained from over 200 trauma and burns patients across the ultra-early (≤1 hour), immediate (4–12 hours) and acute (48–72 hours) post-injury settings. Furthermore, we have examined whether a measurement of total heme levels on day 1 post-burn can distinguish between patients with differing clinical outcomes, specifically non-survival and the development of sepsis. Accompanying these *ex vivo* studies, we have investigated whether pre-conditioning monocytes with heme can induce endotoxin tolerance *in vitro*.

## Materials and methods

### Patient cohorts

#### Burns

This manuscript presents data acquired between November 2016 and September 2023 from adult (≥16 years) burns patients admitted to the West Midlands Regional Burns Centre (WMRBC) within 24 hours of sustaining a total body surface area (TBSA) burn ≥15%. Based at the Queen Elizabeth Hospital Birmingham, the WMRBC is one of three burns centres participating in the Scientific Investigation of the Biological Pathways Following Thermal Injury-2 (SIFTI-2) study, an ongoing prospective, longitudinal observational cohort study of children and adult patients with moderate and severe burn injury. Details relating to study design, exclusion criteria and the procedure of patient consent are described in the SIFTI-2 study protocol (28). The SIFTI-2 study (trial registration number:NCT04693442) received ethical approval from the West Midlands, Coventry and Warwickshire Research Ethics Committee (REC reference:16/WM/0217).

#### Trauma

Data generated from the analysis of peripheral blood samples acquired from subjects enrolled into the Brain Biomarkers after Trauma Study (BBATS) between May 2014 and August 2018 are presented in this manuscript. Conducted at a single Major Trauma Centre site in the UK (University Hospitals Birmingham NHS Foundation Trust (UHBFT), Birmingham), BBATS is an ongoing prospective longitudinal observational study of adult trauma patients. On a 24/7 basis, pre-hospital emergency care teams obtain blood samples from adult trauma patients (≥18 years) with a suspected injury severity score (ISS) ≥8 within 1 hour of injury (defined as the time of phone call to emergency services). Details relating to patient capacity and consent, enrolment and study exclusion criteria have been described previously (2, 29, 30). Ethical approval for the study was granted by the North Wales Research Ethics Committee - West (REC reference:13/WA/0399, Protocol Number: RG_13–164).

### Clinical data collection

Patient and injury details were obtained prospectively from electronic and physical medical records. Data collected included patient age, gender, mechanism of injury, time of injury, severity of injury [Injury Severity Score (ISS), New ISS (NISS) and Glasgow Coma Scale (GCS)], percent TBSA, percent TBSA full thickness, baux score, revised baux score, abbreviated burn severity index, sequential organ failure assessment score and Denver score. Albumin concentrations were measured as part of routine biochemistry investigations during inpatient stays, with the results retrospectively extracted for each patient from the electronic clinical records system used by UHBFT.

### Patient outcomes

Adhering to the recommendations of the 2007 American Burn Association (ABA) consensus for the definition of sepsis and infections in burns patients (31), a diagnosis of sepsis was made when patients met ≥3 of the six sepsis ABA trigger criteria and had a documented infection, identified as either (i) a positive bacterial culture from wound swabs, blood, sputum or urine samples, or (ii) a clinical response to antimicrobials. Data regarding patient mortality and ICU and hospital-free days (calculated as 30 minus the number of days the patient stayed in ICU and hospital respectively) was extracted from electronic clinical records. Patients who died in the hospital or ICU setting within 30 days of admission were assigned a score of 0.

### Blood sampling

Blood samples were collected into BD Vacutainers^®^ (BD Biosciences, UK) containing lithium heparin, z-serum clotting activator or a 1/10 volume of 3.2% trisodium citrate. For patients enrolled into BBATS, blood samples were obtained at three post-injury time-points; pre-hospital (≤1 hour), 4–12 and 48–72 hours. In the pre-hospital setting, blood samples were obtained during the intravenous cannulation of patients or by venepuncture. Vacutainers were stored at room temperature (RT) during transportation to hospital, where, upon arrival, they were stored at 4°C, and collected for analysis within 1 hour by a single researcher on a 24/7 basis. For subjects enrolled into the SIFTI-2 study, data presented in this manuscript were generated from the analysis of blood samples acquired at days 1 and 3 post-burn.

60 adults (mean age 34 years, range 19–69 years) served as a cohort of healthy controls (HC), who we defined as individuals not taking any regular medication for a diagnosed illness and who had not experienced an acute episode of infection prior to enrolment. HC were recruited in accordance with the ethical approval granted by the University of Birmingham Research Ethics Committee (Ref: ERN_12–1184).

### Haematological analysis

Full blood cell counts were performed on citrated or heparinsed anti-coagulated whole blood using the Sysmex XN-1000 haematology analyser (Sysmex UK, Milton Keynes, UK). Instrument performance was ensured by daily internal quality control measurements (XN check, Sysmex UK) and enrolment into a national external quality assurance scheme (UKNEQAS, Watford, UK).

### Preparation of platelet free plasma and serum

PFP was prepared from citrate anti-coagulated whole blood by a two-step centrifugation process. Blood samples were centrifuged at 2,000 × *g* for 20 minutes at 4°C, after which the top two-thirds of platelet-poor plasma (PPP) was carefully removed. PPP was then subjected to centrifugation at 13,000 × *g* for 2 minutes at 4°C to generate PFP, which was collected and stored at −80°C until analysed.

Serum was prepared from blood collected into BD vacutainers containing z-serum clotting activator. Following a 30 minute incubation at RT, samples were centrifuged at 1,620 x g for 10 minutes at 4°C, after which serum was removed and stored at −80°C until analysed.

### Preparation of heme

Prepared fresh on the day of experimentation, heme stock solutions were generated by dissolving 6.5 mg synthetic hemin (Stratech, Cambridge, UK) in dimethyl sulfoxide (Merck, Dorset, UK). Stock solutions and vehicle control were diluted in phosphate buffered saline or RPMI-1640 media supplemented with 2 mM L-glutamine, 100 U/ml penicillin, 100 µg/ml streptomycin (GPS) and 20% fetal calf serum (FCS) prior to cell treatments.

### Cell culture and isolation of PBMCs

Human monocytic THP-1 cells were purchased from the American Type Culture Collection (Virginia, USA) and cultured at 37°C/5%CO_2_ in RPMI-1640 media supplemented with GPS and 10% heat-inactivated FCS [hereafter referred to as complete media (CM)]. PBMCs were isolated from heparin anti-coagulated blood samples by density gradient centrifugation using Ficoll-Paque PLUS media (Cytiva, Sheffield, UK).

### Measurements of heme, hemopexin and haptoglobin

Following manufacturer’s instructions, concentrations of total heme in PFP were determined using a heme assay kit (Abcam, Cambridge, UK). Enzyme linked immunosorbent assays (ELISAs) to quantify concentrations of hemopexin and haptoglobin in PFP were performed in accordance with manufacturer’s protocols (Abcam).

### Cytokine and chemokine measurements

Following manufacturer’s guidelines, serum concentrations of IL-1 receptor antagonist (IL-1Ra), interleukin (IL)-6, IL-10, granulocyte colony stimulating factor (G-CSF) and monocyte chemoattractant protein-1 (MCP-1) were determined using a commercially available magnetic bead multiplex immunoassay (BioRad, Hertfordshire, UK).

### LPS stimulation of whole blood and quantification of TNF-α and interleukin-6 levels

For the *ex vivo* analysis of leukocyte function, 400 µl aliquots of heparinised whole blood were stimulated for 4 or 18 hours (37°C/5%CO_2_) with 10 ng/ml LPS from *Escherichia coli* (serotype 0111:B4; Merck) or vehicle control. Post-treatment, samples were centrifuged at 461 x g for 8 minutes at 4°C, after which supernatants were collected and stored at −80°C until analysed. Following manufacturer’s instructions, TNF-α and IL-6 concentrations were determined using commercially available ELISAs (R and D Systems, Oxford, UK), with results normalised to monocyte count.

### Treatments of THP-1 cells and PBMCs

To examine the role of glycolysis and mitogen activated protein kinase (MAPK) signalling in TNF-α production by monocytes, 1x10^6^ THP-1 cells or PBMCs in CM were treated for 1 hour (37°C/5%CO_2_) with 5–50 mM 2-Deoxy-D-Glucose (2-DG; Merck), 10 µM PD98059 (Cell Signalling Technology (CST) Leiden, Netherlands) or vehicle control, after which cells were stimulated for 4 hours with 100 ng/ml or 1 µg/ml LPS respectively at 37°C/5%CO_2_. Post-incubation, samples were centrifuged (1,500 x g, 2 minutes), and cell free supernatants collected for the determination of TNF-α concentrations by ELISA.

To investigate the effect of heme treatment on monocyte function, THP-1 cells (1–2x10^6^) or PBMCs (1x10^6^), resuspended in RPMI media supplemented with GPS (hereafter referred to as assay media) or RPMI + GPS + 20% FCS, were cultured for 1 or 4 hours (37°C/5% CO_2_) in the presence of 10–20 µM heme or vehicle control. Post-treatment, cells were pelleted (1,500 x g, 2 minutes) and resuspended in CM or assay media prior to a 30 minute, 2 hour or 4 hour stimulation with 100 ng/ml or 1 µg/ml LPS or vehicle control (37°C/5%CO_2_). Post-culture, samples were centrifuged (1,500 x g, 2 minutes), and either cell free supernatants collected for the determination of TNF-α and lactate concentrations, or cell pellets resuspended in RLT or SDS lysis buffer in preparation for RNA isolation and Western blotting respectively.

To ascertain whether serum collected from thermally-injured patients could modulate monocyte function, THP-1 cells (0.5–1x10^6^) were cultured for 24 hours (37°C/5%CO_2_) in assay media supplemented with 10% sera pooled from 5 burns patients on day 1 of injury or HC. Post-treatment, cells were stimulated for 2 or 4 hours with 1 µg/ml LPS (37°C/5%CO_2_), after which cells were pelleted (1,500 x g, 2 minutes) and either cell free supernatants collected for the determination of TNF-α concentrations or cell pellets resuspended in RLT lysis buffer in preparation for RNA isolation. Endotoxin concentrations in patient and HC serum samples were determined using a commercially available limulus amebocyte lysate chromogenic endpoint assay (Hycult Biotech, Pennsylvania, USA).

### Measurements of lactate concentration and lactate dehydrogenase activity in cell-free supernatants

In accordance with manufacturer’s guidelines, lactate concentrations and LDH activity in 5–10 µl aliquots of cell-free supernatants collected from heme and/or LPS treated THP-1 cells or PBMCs were determined using commercially available lactate or LDH activity assay kits (Merck). For the LDH activity assay, a positive control was generated by treating 1x10^6^ THP-1 cells with 10 µM staurosporine (Merck) for 4 hours (37°C/5% CO_2_).

### Western blotting

Cell lysates prepared from 1 µg/ml LPS stimulated THP-1 cells pre-treated for 4 hours with 20 µM heme or vehicle control were separated on 10% SDS-polyacrylamide gels. Following protein transfer to polyvinylidene difluoride membranes (Bio-Rad, Hertfordshire, UK), blots were probed overnight at 4°C with rabbit anti-human antibodies (purchased from CST) directed against phosphorylated NF-κB p65 (Ser536) or ERK1/2 (Thr202/Tyr204). Post-incubation, membranes were washed in tris-buffered saline containing 0.001% tween (TBST) and incubated for 1 hour at RT with a goat anti-rabbit secondary antibody conjugated to horse radish peroxidase (HRP; diluted 1:4000 in TBST; GE Healthcare, Buckinghamshire). HRP activity was detected using enhanced chemiluminescence (Cytiva, Massachusetts, USA). To confirm equal loading of proteins, blots were stripped with harsh stripping buffer (Abcam) and probed with antibodies against total P65 or ERK1/2 (CST; diluted 1:1000 in TBST). Densitometry analysis was performed using Image J software (National Institute of Health, Maryland, USA).

### Real time polymerase chain reaction

Following manufacturer’s guidelines, total RNA was extracted from THP-1 cells or PBMCs using an RNeasy Mini kit (Qiagen Ltd) or TRIzol™ reagent (ThermoFisher Scientific UK, Chesire, UK), with concentrations quantified using a NanoDrop 2000 (ThermoFisher Scientific).

mRNA expression levels of TNF-α and HO-1 were determined, relative to 18S, by RT-PCR using the iTaq^™^ Universal SYBR^®^ Green One-step kit mastermix (Bio-Rad), 5 ng total RNA and primers (TNF-α: Forward 5’CCT CTC TCT AAT CAG CCC TCT G3’, Reverse 5’GAG GAC CTG GGA GTA GAT GAG3’. HO-1: Forward 5’CAG GAT TTG TCA GAG GCC CTG AAG G3’, Reverse 5’TGT GGT ACA GGG AGG CCA TCA CC3’. 18S: Forward 5’GTA ACC CGT TGA ACC CCA TT3’, Reverse 5’CCA TCC AAT CGG TAG CG3’). All reactions had a total volume of 5 µl and were performed in triplicate. Data were acquired using a Bio-Rad sfx cycler (Bio-Rad) and analysed by the 2^-ΔΔCt^ method using Bio-Rad CFX manager software (Bio-Rad). Gene expression was calculated relative to 18S. For cell treatment experiments, results are presented as fold change above untreated controls.

### Statistical analyses

Statistical analyses were performed using GraphPad PRISM software (GraphPad Software Ltd, California, USA) and R v4.2.2 (R Foundation for Statistical Computing, Vienna, Austria). Data distribution was assessed using the Kolmogorov-Smirnov or Shapiro-Wilk normality tests. Data that followed a normal distribution were analysed using a one-way ANOVA with either a Dunnett or Bonferroni multiple comparison *post-hoc* test, an unpaired student t test or a paired student t test. To analyse non-normally distributed data, a Kruskal-Wallis test with Dunn’s multiple comparison *post-hoc* test, Mann-Whitney U test or Wilcoxon matched-pairs signed rank test was performed. Relationships between continuous variables were assessed using a Spearman’s correlation. For comparisons of continuous variables between survivors and non-survivors, and septic and non-septic patients, Mann-Whitney U tests or independent samples t tests were performed, whilst Chi-squared tests were conducted to compare categorical variables. Box and whisker plots are presented in Tukey style. The threshold for statistical significance was set at p≤ 0.05.

Associations between concentration levels in predictor variables and outcomes were modelled using logistic regression models. Initially, concentration is the only covariate included in the models (the unadjusted models). Further models, adjusted for age, gender, and TBSA are also fit to the data. Odds ratios, 95% CIs and p-values are reported for all models. Predicted probabilities, and 95% CIs, of outcomes are reported for the unadjusted models. The potential discriminatory ability of concentrations measured on day 1 to distinguish between survivors and non survivors was assessed through fitting logistic regression models. Discriminatory performance of the models is reported using area under the receiver-operating characteristic curve (AUROC) and Brier scores.

## Results

### Patient demographics

A total of 98 thermally-injured patients were included in this study (**Table 1**). Patients had a mean age of 47 years (range 16–84 years), and presented with a mean TBSA burn of 35% (range 15–85%). Flame burn was the predominant mechanism of injury, with 43% of the cohort sustaining an inhalation injury. The incidence of sepsis and mortality was 53% and 20% respectively, with day 6 post-burn the median time to first septic episode (**Table 1**).

**Table 1:** Burns patients demographics.

Characteristic	Burns patients (n=98)
Age, years (range)	47 (16–84)
Gender, (M:F)	76:22
% TBSA (range)	35 (15–85)
% FT TBSA (range)	19 (0–80)
Inhalation injury (Y:N)	42:56
Mechanism of injury	
Flash, n (%) Flame, n (%) Flame and flash, n (%) Electrical, n (%) Scald, n (%)	7 (7) 81 (83) 5 (5) 1 (1) 4 (4)
ABSI (range)	7 (2–14)
Baux (range)	83 (34–143)
rBaux (range)	90 (39–160)
Day 1 SOFA (range)	6 (0–17)
Day 1 Denver (range)	2 (0–7)
ICU free days (range)	16 (0–30)
Hospital free days (range)	4 (0–24)
Sepsis (Y:N)	46:40^#^
Mortality (Y:N)	19:76^@^

Data are expressed as mean (range) unless otherwise stated.

^#^Due to morality within 7 days of hospital admission from non-septic causes, or loss to clinical follow up, sepsis status was not determined for 12 patients.

^@^Due to loss to clinical follow up, mortality status was not recorded for 3 patients.

ABSI, Abbreviated burn severity index; ICU, Intensive care unit; SOFA, Sequential organ failure assessment; TBSA, Total body surface area.

Road traffic collisions (53.1%) were the predominant mechanism of injury in our cohort of 147 adult trauma patients who had a mean age of 42 years (range 18–95 years) and a mean ISS of 25 (range 9–66) (**Table 2**). The mean time of pre-hospital blood sampling was 42 minutes post-injury (range 13–60 minutes).

**Table 2:** Trauma patients demographics.

Characteristic	Trauma patients (n=147)
Age, years (range)	42 (18–95)
Gender, (M:F)	126:21
Time to pre-hospital sample, minutes post-injury (range)	41 (13–60)
ISS (range)^#^	25 (9–66)
NISS (range)^#^	37 (9–75)
Admission GCS score (range)	10 (3–15)
Mechanism of injury	
*Fall, n (%)* *A/P, n (%)* *Blunt, n (%)* *RTC, n (%)*	30 (20) 33 (22) 6 (5) 78 (53)
ICU-free days (range)	19 (0–30)
Hospital-free days (range)	7 (0–29)
Mortality, n (%)	24 (16.3)

Data are expressed as mean (range) unless otherwise stated.

^#^Information relating to ISS and NISS was available for 137 patients. A/P, Assault/Penetrating;

GCS, Glasgow coma scale; ICU, Intensive care unit; ISS, Injury severity score;

NISS, New injury severity score; RTC, Road traffic collision.

### Severe thermal injury results in an immediate and sustained systemic inflammatory response that is accompanied by impaired *ex vivo* LPS-induced pro-inflammatory cytokine production by whole blood leukocytes

**Figure 1:**
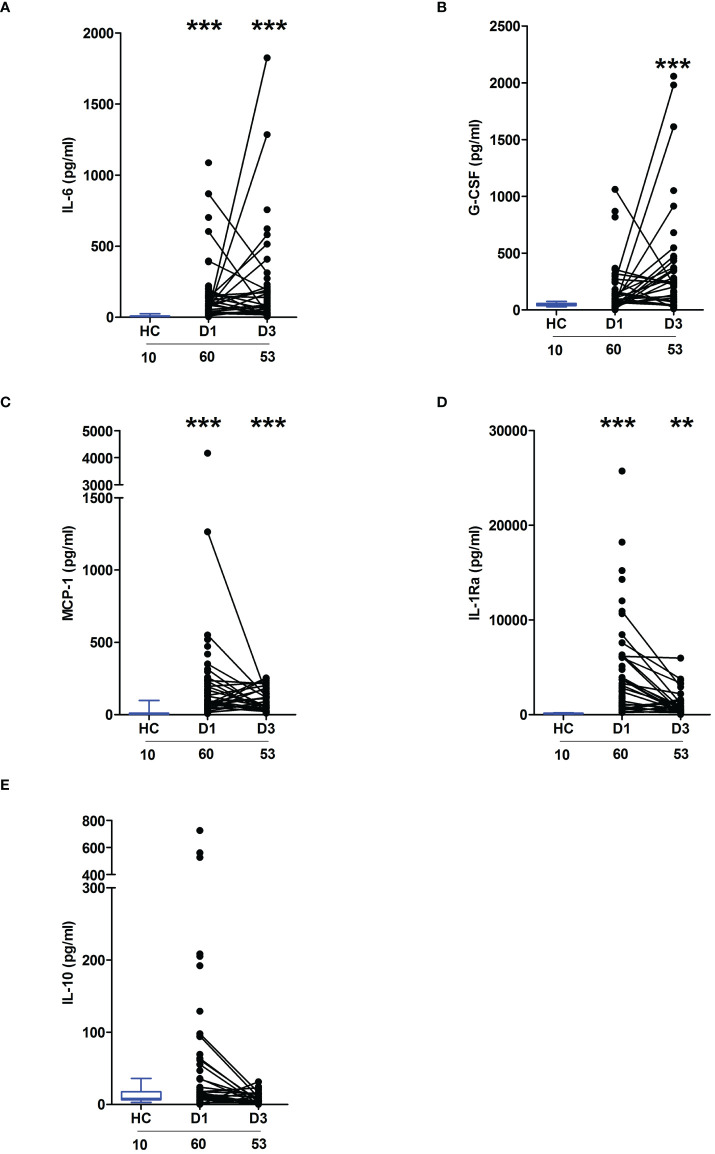
Severe thermal injury results in an immediate and sustained systemic inflammatory response. **(A–E)** Comparison of concentrations of interleukin (IL)-6 **(A)**, Granulocyte colony stimulator-factor (G-CSF) **(B)**, Monocyte chemoattractant protein-1 (MCP-1) **(C)**, IL-1 receptor antagonist (IL-1Ra) **(D)** and IL-10 **(E)** in serum samples obtained from thermally-injured patients at days 1 and 3 post-burn and healthy controls (HC). The number of samples analysed are stated below each study time-point. **p<0.005, ***p<0.0005.

In line with our previous work in the setting of major trauma (2), analysis of serum samples obtained from thermally-injured patients revealed an early and persistent elevation in the circulating concentrations of inflammatory cytokines and chemokines, with the levels of IL-6, G-CSF, MCP-1 and IL1-Ra significantly elevated on day 1 and/or day 3 post-burn when compared to the concentrations measured in HC (**Figures 1A–D**). Thermal injury did not alter the circulating levels of IL-10 (**Figure 1E**).

**Figure 2:**
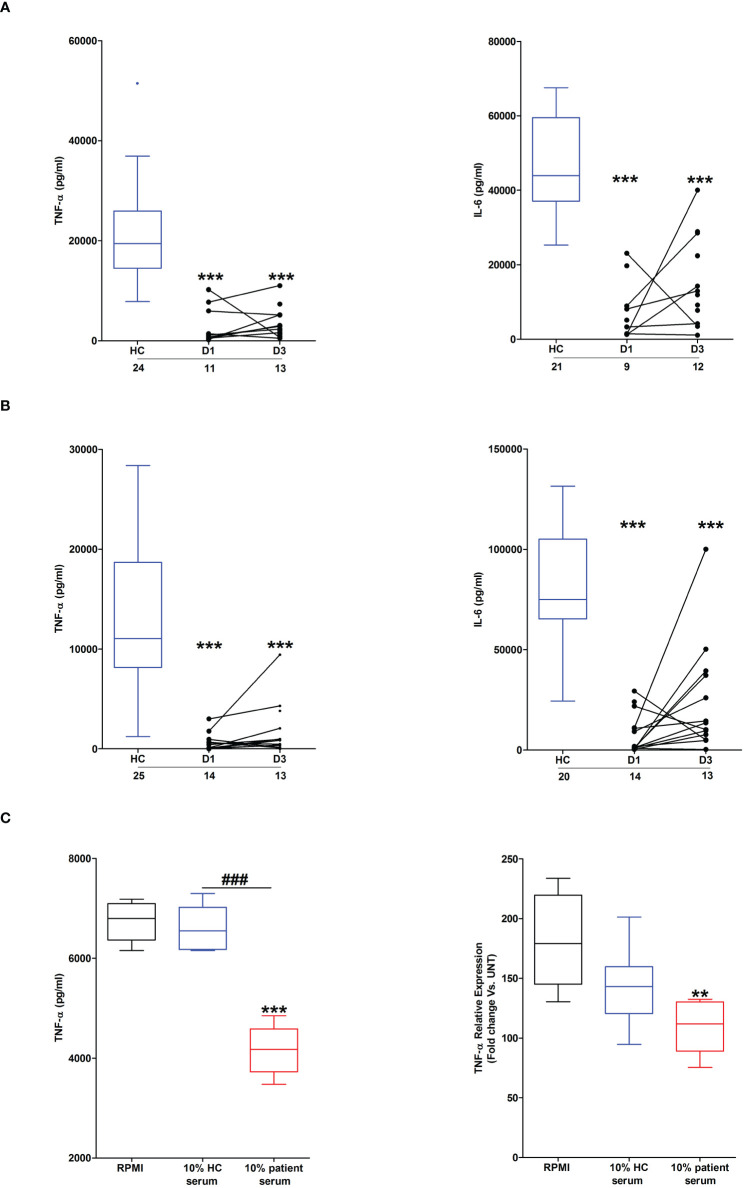
Effect of severe thermal injury on lipopolysaccharide (LPS)-induced pro-inflammatory cytokine production by whole blood leukocytes. **(A, B)** Tumour necrosis factor-alpha (TNF-α; left panel) and interleukin-6 (IL-6; right panel) concentrations measured in supernatants of whole blood samples obtained from burns patients at days 1 and 3 post-injury and healthy controls (HC) following a 4 hour **(A)** or 18 hour **(B)** *ex vivo* stimulation with 10 ng/ml LPS. The number of samples analysed are stated below each study time-point. **(C)** Following a 24 hour culture in media supplemented with 10% serum obtained from burns patients on day 1 of injury or HC (n=5), THP-1 cells were stimulated with 1 µg/ml LPS, after which TNF-α concentrations in cell free supernatants (left panel) or TNF-α mRNA levels (right panel) were measured respectively. For the measurement of TNF-α concentrations in culture supernatants, THP-1 cells were stimulated for 4 hours with LPS. TNF-α mRNA levels were examined in THP-1 cells following a 2 hour stimulation with LPS. **p<0.005, ***p<0.0005 Vs vehicle. ^###^p<0.0005.

Across the ultra-early (<1 hour) and acute (2–72 hours) post-injury phase, we and others, have previously demonstrated that whole blood leukocytes from major trauma patients exhibit impaired *ex vivo* pro-inflammatory cytokine production upon LPS stimulation (1, 2). To determine whether thermal injury also resulted in peripheral endotoxin tolerance, whole blood leukocytes isolated from severe burns patients at days 1 and 3 post-injury were stimulated with LPS for 4 or 18 hours, after which the levels of pro-inflammatory cytokines in cell free supernatants were measured. Compared to samples from HC, significantly lower concentrations of TNF-α and IL-6 were detected in supernatants of LPS-challenged blood from burns patients at both post-injury time-points (**Figures 2A, B**).

Suggesting a role for circulating factors in promoting post-burn endotoxin tolerance, THP-1 cells cultured for 24 hours in media supplemented with 10% serum obtained from thermally-injured patients exhibited impaired TNF-α production and transcription upon LPS stimulation (**Figure 2C**). Compared to HC, endotoxin levels were significantly lower in serum samples of burns patients (**Supplementary Figure 1**).

### Major thermal and traumatic injury results in elevated circulating concentrations of heme that are positively associated with systemic inflammation

**Figure 3:**
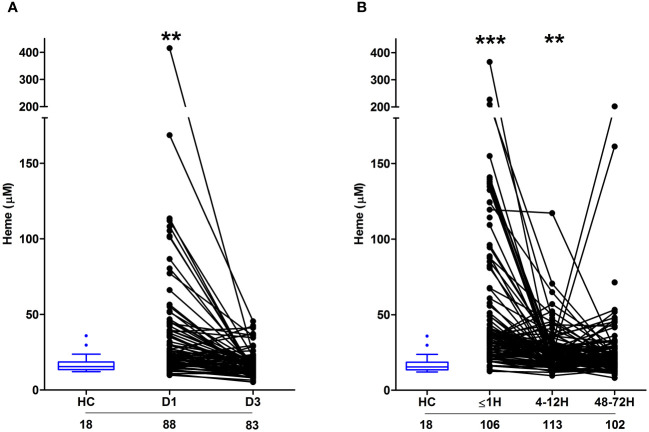
Severe burns and major traumatic injury result in an immediate elevation in plasma concentrations of total heme. **(A, B)** Concentrations of total heme measured in PFP samples of severe burns **(A)** and major trauma **(B)** patients across the ultra-early (≤1H) and acute (4–72H) post-injury setting. The number of samples analysed are stated below each study time-point. **p<0.005, ***p<0.0005. HC, Healthy control.

As reported in **Figure 3A**, relative to HC, significantly higher concentrations of total heme were detected in PFP samples obtained from burns patients on day 1 of injury. At day 3 post-burn, no significant difference was detected in the circulating concentrations of total heme between patients and HC (burns patients, 17.03 ± 0.92 µM Vs HC, 17.59 ± 1.47 µM; p= n.s). Analysis of day 1 samples revealed total heme concentrations were positively associated with % TBSA (r(n=88)=0.456, p=<0.0001), % full thickness TBSA (r(n=88)=0.466, p=<0.0001), baux score (r(n=88)=0.350, p=0.0008) and revised baux score (r(n=88)=0.390, p=0.0002). A negative association was found between total heme levels and the time of sample acquisition post-burn (r(n=88)=-0.411, p=<0.0001). With our patient cohort ranging in age from 16–84 years, we examined whether a relationship existed between circulating heme levels and age. We found no association between patient age and plasma concentrations of heme at days 1 or 3 post-burn (**Supplementary Table 1**).

In our cohort of trauma patients, total heme concentrations were significantly elevated in PFP prepared from blood samples obtained ≤1 and 4–12 hours post-injury when compared to HC (**Figure 3B**). As observed with burns patients, heme levels negatively correlated with the time of sample acquisition post-injury (r(n = 321) =-0.452, p=<0.0001). Analysis of samples acquired within 1 hour of trauma revealed no relationship between heme concentrations and injury severity when assessed using either ISS (r(n = 101) =0.09, p=0.350) or NISS (r(n = 101) =0.130, p=0.194). An examination of the relationship between patient age and heme levels revealed a weak positive association between these two variables only at the 4–12 hour post-injury sampling time-point (**Supplementary Table 1**).

On day 1 of burn injury, we found circulating levels of IL-10 and MCP-1 were positively associated with heme concentrations (**Table 3**). At day 3 post-burn, plasma concentrations of heme positively correlated with the circulating levels of IL1-Ra and MCP-1 (**Table 3**). In our cohort of major trauma patients, in whom we have previously measured levels of circulating pro and anti-inflammatory cytokines (2), we found positive associations between plasma heme levels and the concentrations of IL1-Ra, IL-6, IL-10 and G-CSF at the 4–12 hour post-injury sampling time-point, and between heme and MCP-1 48–72 hours post-injury (**Table 3**).

**Table 3:** Correlative analyses examining the relationship between plasma heme levels and the circulating concentrations of pro and anti-inflammatory cytokines in severe burns and major trauma patients.

	Day 1	Day 3	<1H	4–12H	48–72H
Burns patients					
*IL1-Ra*	R= 0.262 (-0.017–0.503) p= 0.058 n= 53	**R= 0.387** **(0.113–0.606)** **p= 0.006** **n= 50**	N/A	N/A	N/A
*IL-6*	R= 0.219 (-0.063–0.468) p= 0.116 n= 53	R= 0.275 (-0.013–0.520) p= 0.054 n= 50	N/A	N/A	N/A
*IL-10*	**R= 0.668** **(0.479–0.798)** **p= <0.0001** **n= 53**	R= 0.245 (-0.045–0.496) p= 0.087 n= 50	N/A	N/A	N/A
*G-CSF*	R= 0.108 (-0.176–0.374) p= 0.444 n= 53	R= 0.222 (-0.069–0.478) p= 0.121 n= 50	N/A	N/A	N/A
*MCP-1*	**R= 0.383** **(0.118–0.597)** **p= 0.005** **n= 53**	**R= 0.315** **(0.032–0.552)** **p= 0.026** **n= 50**	N/A	N/A	N/A
Trauma patients					
*IL1-Ra*	N/A	N/A	R= 0.113 (-0.126–0.339) p= 0.339 n= 74	**R= 0.355** **(0.126–0.548)** **p= 0.002** **n= 71**	R= 0.120 (-0.140–0.363) p= 0.351 n= 63
*IL-6*	N/A	N/A	R= 0.069 (-0.169–0.299) p= 0.558 n= 74	**R= 0.248** **(0.009–0.461)** **p= 0.037** **n= 71**	R= 0.202 (-0.055–0.435) p= 0.112 n= 63
*IL-10*	N/A	N/A	R= -0.173 (-0.392–0.065) p= 0.141 n= 74	**R= 0.330** **(0.098–0.528)** **p= 0.005** **n= 71**	R= 0.134 (-0.125–0.376) p= 0.296 n= 63
*G-CSF*	N/A	N/A	R= -0.100 (-0.326–0.140) p= 0.407 n= 74	**R= 0.282** **(0.045–0.489)** **p= 0.017** **n= 71**	R= 0.147 (-0.112–0.388) p= 0.250 n= 63
*MCP-1*	N/A	N/A	R= 0.110 (-0.129–0.336) p= 0.352 n= 74	R= 0.186 (0.121–0.408) p= 0.121 n= 71	**R= 0.252** **(-0.004–0.476)** **p= 0.047** **n= 63**

95% confidence intervals are presented in paratheses.

Significant associations according to Spearman’s rank correlation coefficient are indicated in bold font.

N/A, not applicable.

### Fragmented red cells and damaged tissue are potential sources of circulating heme in major burns and trauma patients

**Figure 4:**
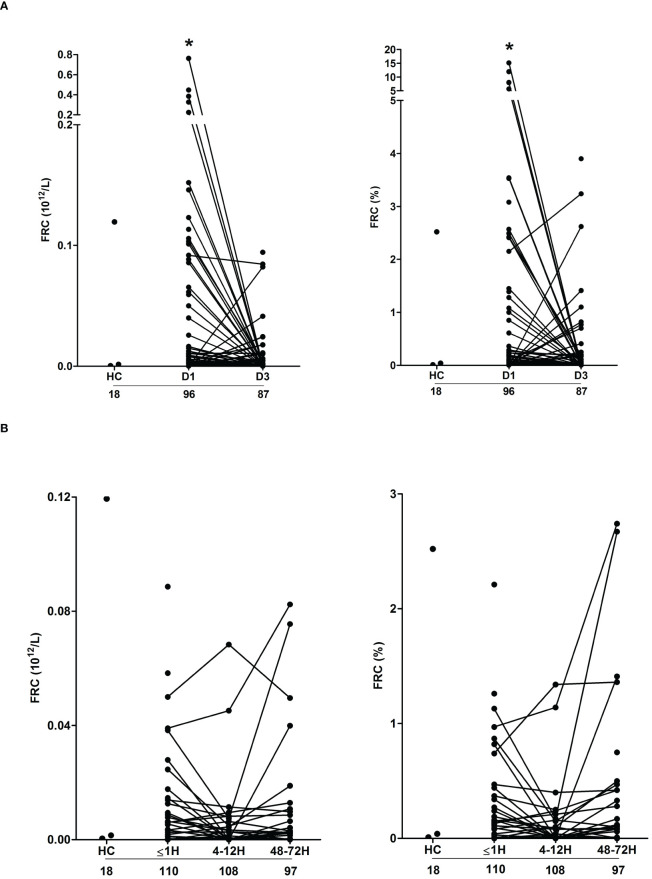
Severe thermal injury but not major trauma results in the generation of fragmented red blood cells. **(A, B)** Absolute number (left panel) and frequency (right panel) of fragmented red blood cells (FRC) in peripheral blood samples of burns patients on days 1 and 3 post-injury **(A)** and major trauma patients **(B)** in the ultra-early (≤1H) and acute (4–72H) post-injury setting. The number of samples analysed are stated below each study time-point. *p<0.05. HC, Healthy control.

Confirming previous observations (19), thermally-injured patients presented on day 1 of injury with significantly elevated counts and frequencies of FRC, with readings returning to levels comparable to those recorded for HC by day 3 post-burn (**Figure 4A**). Both the absolute number and frequencies of FRC on day 1 of burn injury were positively associated with total heme concentrations (absolute number, r(n=88)=0.472, p= <0.0001; frequency, r(n=88)=0.471, p=<0.0001).

Compared to HC, we detected no difference in the absolute number or frequency of FRC in blood samples obtained from trauma patients at any of our three study time-points (**Figure 4B**). Used as a marker of tissue injury, we previously reported that circulating concentrations of G-actin are elevated in the immediate post-injury setting (29). Analysis of plasma samples acquired from trauma patients ≤1 hour post-injury revealed a significant positive relationship between total heme levels and the circulating concentrations of G-actin (r(n=37)=0.366, p=0.02).

### Severe thermal and major traumatic injury results in the depletion of plasma proteins involved in heme and haemoglobin scavenging

**Figure 5:**
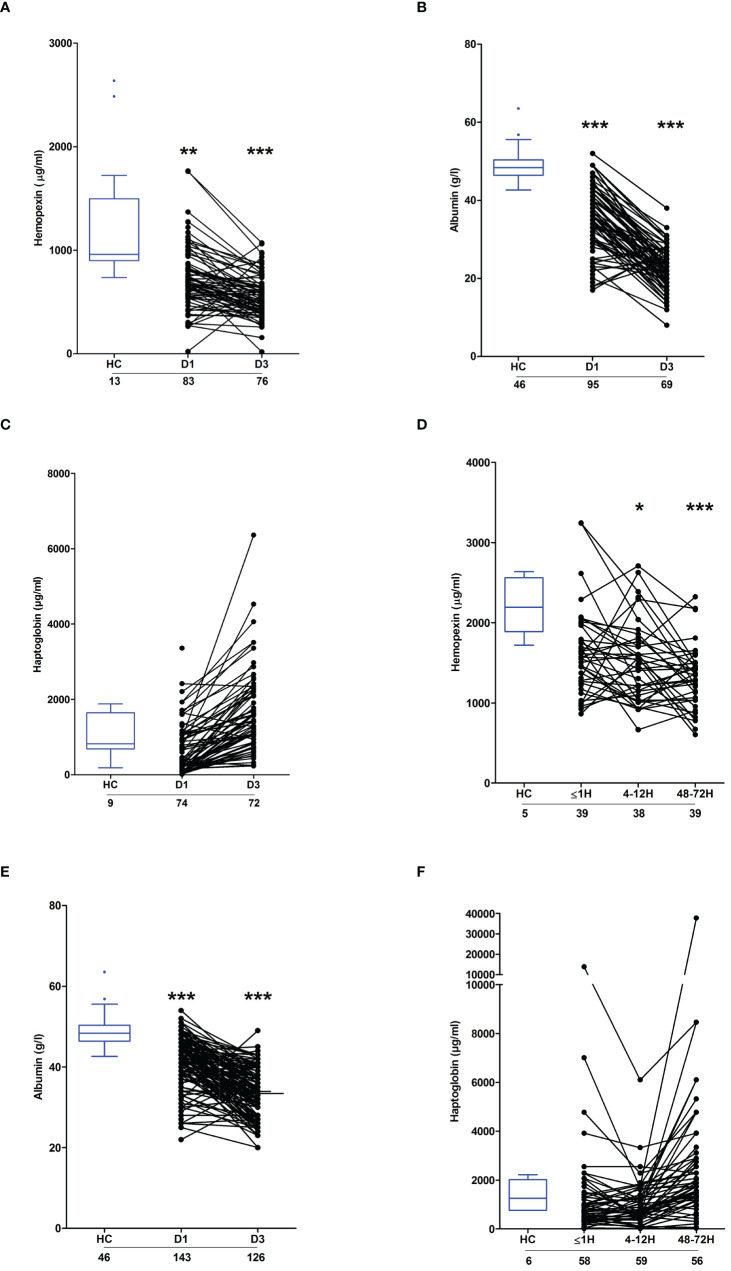
Effect of severe thermal injury and major trauma on the concentrations of plasma proteins implicated in heme and haemoglobin scavenging. **(A‐C)** Concentrations of hemopexin **(A)**, albumin **(B)** and haptoglobin **(C)** measured in PFP samples acquired from thermally-injured patients on days 1 and 3 post-burn. **(D‐F)** Concentrations of hemopexin **(D)**, albumin **(E)** and haptoglobin **(F)** measured in PFP samples acquired from major trauma patients during the ultra-early (≤1H) and acute (4–72H) post-injury phase. The number of samples analysed are stated below each study time-point. *p<0.05, **p<0.005, ***p<0.0005. HC, Healthy control.

Circulating heme levels are regulated by a scavenging system comprised of the plasma proteins hemopexin, haptoglobin and albumin (23, 24). Burns patients presented with significantly lower concentrations of hemopexin and albumin at days 1 and 3 post-injury (**Figures 5A, B**). Repeated measures analysis revealed a significant decrease in the concentrations of both proteins between the two study time-points (**Supplementary Figure 2**). On day 1 of burn injury, significant negative associations were found between % full thickness TBSA and the plasma levels of hemopexin (r(n=83)=-0.310, p=0.004) and albumin (r(n =95) =-0.404, p=<0.0001). Thermal injury had no effect upon the circulating concentrations of haptoglobin (**Figure 5C**).

Relative to HC, hemopexin levels were significantly lower in PFP samples obtained from trauma patients 4–12 and 48–72 hours post-injury (**Figure 5D**). Similarly, on days 1 and 3 post-trauma, patients presented with significantly reduced plasma concentrations of albumin (**Figure 5E**). Traumatic injury did not alter the circulating levels of haptoglobin (**Figure 5F**).

### Major traumatic injury but not severe burns lead to an up-regulation in HO-1 gene expression in PBMCs

**Figure 6:**
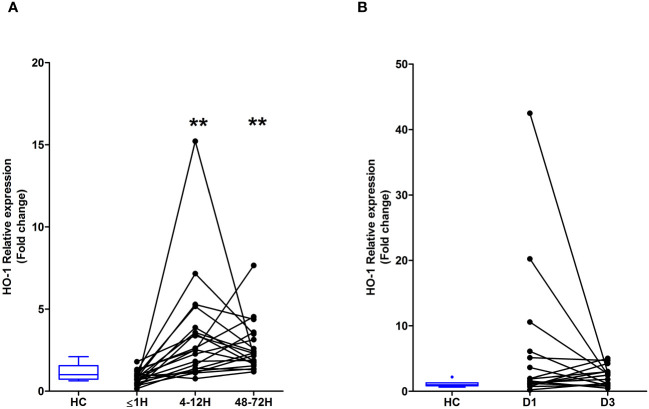
Major traumatic injury but not severe burns results in increased heme oxygenase-1 (HO-1) gene expression in peripheral blood mononuclear cells (PBMCs). **(A)** Comparison of HO-1 mRNA levels in PBMCs of healthy controls (HC; n=10) and major trauma patients (n=20) at three post-injury time-points. **(B)** HO-1 gene expression in PBMCs isolated from HC (n=8) and severe burns patients (n=16) at days 1 and 3 post-thermal injury. **p<0.005.

HO-1 is a stress-inducible enzyme that catalyses the degradation of intracellular heme (24). Compared to HC, HO-1 gene expression was significantly increased in PBMCs isolated from major trauma patients 4–12 and 48–72 hours post-injury (**Figure 6A**). In contrast, we detected no difference in HO-1 mRNA levels between PBMCs isolated from burns patients at days 1 or 3 post-injury and HC (**Figure 6B**).

### Elevated total heme concentrations are associated with poor clinical outcomes post-burn

To investigate whether elevated plasma concentrations of total heme on day 1 of thermal injury were associated with poor clinical outcomes, we performed exploratory analysis to examine if any associations existed between total heme levels and either patient mortality or the development of sepsis. Within our cohort of 98 burns patients, 4 died within 7 days of injury from non-septic causes and 8 withdrew from clinical follow up during their in-hospital stay. Of the remaining 86 patients, 7 did not have a measurement of total heme levels on day 1 of injury. Thus, sepsis status was determined for 79 patients, with 42 developing this secondary complication, a prevalence rate of 53%. The demographics of our septic and non-septic cohorts are summarised in **Supplementary Table 2**. Of the 95 patients for whom survival status was determined, 7 did not have a measurement of total heme levels on day 1 of injury. Mortality was therefore assessed in 88 patients, with 14 patients meeting this clinical endpoint. The demographic and clinical data of survivors and non-survivors are summarised in **Supplementary Table 2**.

**Figure 7:**
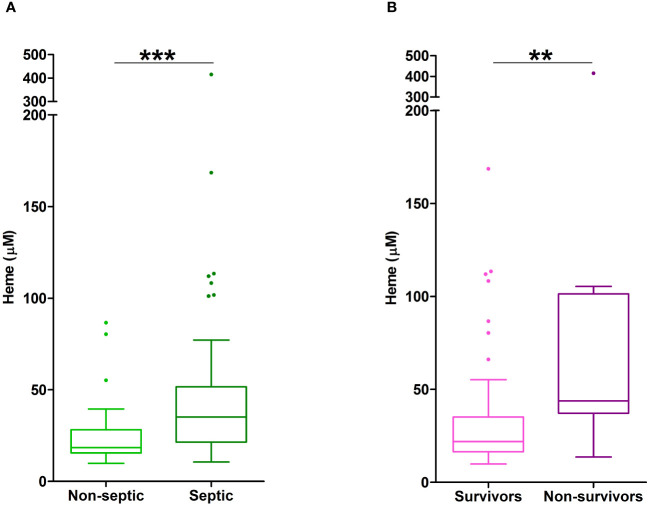
Elevated plasma concentrations of total heme are associated with poor clinical outcomes in severe burns patients. **(A)** Total heme concentrations measured in PFP samples obtained on day 1 of injury from thermally-injured patients who did (n=42) or did not (n=37) develop sepsis post-burn. **(B)** Comparison of day 1 total heme levels in PFP samples of survivors (n=74) and non-survivors (n=14) of severe thermal injury. **p<0.005, ***p<0.0005.

Non-survivors of thermal injury and patients who developed sepsis post-burn presented on day 1 of injury with significantly elevated plasma concentrations of total heme (**Figures 7A, B**). To investigate this relationship further, we calculated the predicted probabilities of mortality and the development of sepsis in burns patients at days 1 and 3 post-injury using the quantiles of heme concentrations measured at these two sampling time-points (**Table 4**).

**Table 4:** Predicted probabilities of mortality and the development of sepsis in thermally-injured patients based on the quantiles of total heme concentrations measured on days 1 and 3 post-burn.

	Heme concentration (µM)	Probability of Death (% (95% CI))	Probability of Sepsis (% (95% CI))
**Day 1**	10	0.6% (0% to 8.1%)	52.4% (36.7% to 67.6%)
	17	1.4% (0.2% to 9.9%)	51.8% (36.5% to 66.9%)
	24	3.1% (0.7% to 12.6%)	51.7% (30.4% to 72.4%)
	39	10.4% (4.3% to 23.2%)	52.3% (29.2% to 74.5%)
	415	75.6% (4% to 99.6%)	71.7% (0% to 100%)
**Day 3**	5	8.8% (3.1% to 22.7%)	31.4% (16.3% to 51.8%)
	12	11.7% (5.7% to 22.5%)	42.3% (29.9% to 55.7%)
	14	12.7% (6.6% to 22.8%)	45.6% (34% to 57.7%)
	20	16% (9.3% to 26.2%)	55.6% (42.9% to 67.7%)
	45	37% (9.3% to 77%)	87.1% (51.7% to 97.7%)

In unadjusted logistic regression models, we found that a difference of 6.5 µM in the circulating concentration of total heme corresponded to a relative increase in the odds of sepsis of 24% (OR, 1.24 (95% CI, 1.05, 1.46), p=0.013). However, when models were adjusted for age, gender and % TBSA, the size of this association was reduced (OR, 1.12 (95% CI, 0.95, 1.33), p=0.172). In terms of mortality, a difference of 6.5 µM in the circulating concentration of total heme corresponded to a relative increase in the odds of mortality of 63% (OR, 1.63 (95% CI, 1.12, 2.37), p=0.004), an association that remained in a model adjusted for age, gender and % TBSA (OR, 1.52 (95% CI, 1.02, 2.28), p=0.021).

To assess the potential discriminatory ability of day 1 total heme levels to distinguish between survivors and non survivors, prognostic models were examined. AUROC analyses revealed day 1 total heme levels had moderate power to discriminate between these two patient groups (AUROC, 0.768 (95% CI, 0.615–0.922), Brier Score 0.119). In comparison, a model built on rBAUX scores generated an AUROC value of 0.718 (95% CI, 0.587–0.848; Brier score 0.123).

### Exposure to heme induces endotoxin tolerance in monocytes

Suggesting a link between elevated heme concentrations and monocyte endotoxin tolerance post-burn, significant negative associations were detected between circulating total heme levels on day 1 of injury and the concentrations of TNF-α and IL-6 measured in supernatants of whole blood samples challenged *ex vivo* with LPS for 4 hours (TNF-α, r(n=11)=-0.736, p=0.013; IL-6, r(n=9)=-0.717, p=0.037) or 18 hours (IL-6, r(n=14)=-0.582, p=0.029).

**Figure 8:**
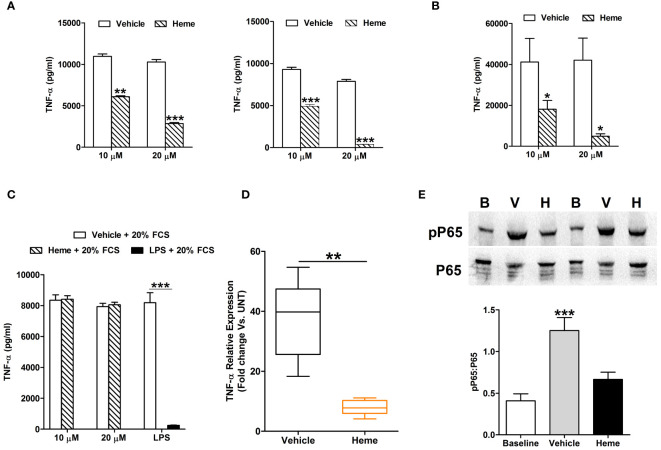
*In vitro* heme treatment induces endotoxin tolerance in human monocytes. **(A)** Concentration of tumour necrosis factor-alpha (TNF-α) detected in supernatants of lipopolysaccharide (LPS) challenged THP-1 cells (n=6) pre-treated for 1 hour (left panel) or 4 hours (right panel) with 10 or 20 µM heme. **p<0.005, ***p<0.0005. **(B)** Concentration of TNF-α measured in supernatants of LPS challenged PBMCs (n=8) pre-treated for 4 hours with 10 or 20 µM heme. *p<0.05. **(C)** TNF-α levels recorded in supernatants of LPS challenged THP-1 cells pre-treated for 4 hours with 20 µM heme (n=8) or 100 ng/ml LPS (n=3) in media supplemented with 20% fetal calf serum (FCS). **(D)** Comparison of TNF-α mRNA levels in LPS stimulated THP-1 cells pre-treated for 4 hours with 20 µM heme or vehicle control (n=5). **(E)** Representative Western blot (top panel) and collated densitometry data (bottom panel, n=7) showing the phosphorylation status of the NF-κB subunit P65 in LPS challenged THP-1 cells pre-treated for 4 hours with 20 µM heme or vehicle control. **p<0.005 Vs. Baseline. B, Baseline; V, vehicle control; H, heme-treated.

*In vitro* exposure to heme did not trigger pro-inflammatory cytokine production by THP-1 monocytes (**Supplementary Figure 3A**). However, when compared to vehicle controls, THP-1 cells pre-treated for 1 or 4 hours with 10 or 20 µM heme generated significantly less TNF-α upon subsequent LPS challenge (**Figure 8A**), a finding we confirmed in primary human monocytes (**Figure 8B**). Demonstrating the specificity of this response, heme-induced, but not LPS-induced, endotoxin tolerance was prevented by culturing THP-1 cells in media supplemented with 20% FCS (**Figure 8C**). An assessment of cellular toxicity found no difference in the activity of lactate dehydrogenase (LDH) in supernatants derived from vehicle or heme treated THP-1 cells (**Supplementary Figure 3B**). Staurosporine treatment served as a positive control in this assay and resulted in significantly increased LDH activity in culture supernatants (**Supplementary Figure 3B**).

Analysis of gene expression found that, relative to vehicle treated controls, TNF-α mRNA levels were significantly lower in LPS challenged THP-1 cells pre-treated with heme (**Figure 8D**). This heme-induced impairment in TNF-α gene transcription was associated with reduced phosphorylation of the NF-κB subunit P65 (**Figure 8E**).

### Heme pre-treatment results in impaired LPS-induced activation of the MAPK ERK1/2

**Figure 9:**
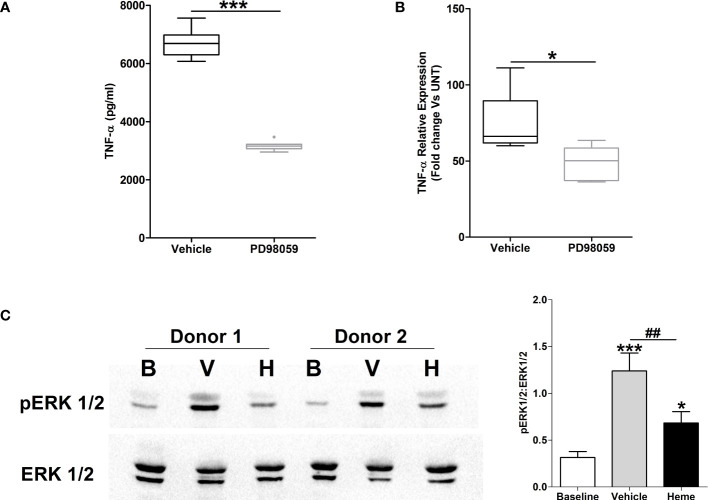
Impaired lipopolysaccharide (LPS)-induced activation of the MAPK extracellular signal regulated kinase 1/2 (ERK 1/2) in heme pre-treated THP-1 cells. **(A, B)** Following a 1 hour pre-treatment with 10 µM PD98059 or vehicle control, THP-1 cells were stimulated with 1 µg/ml LPS, after which tumour necrosis factor-alpha (TNF-α) concentrations in culture supernatants (n=10) **(A)** and mRNA expression (n=6) **(B)** was measured. *p<0.05, ***p<0.0005. **(C)** Representative Western blot (left panel) and collated densitometry data (right panel, n=6) showing the phosphorylation status of ERK1/2 in LPS challenged THP-1 cells pre-treated for 4 hours with 20 µM heme or vehicle control. *p<0.05, ***p<0.0005 Vs Baseline. ^##^p<0.005. B, Baseline; V, vehicle control; H, heme-treated.

Activation of the MAPK ERK1/2 has been implicated in the production of TNF-α by LPS stimulated monocytes (32, 33). In line with this data, THP-1 monocytes pre-treated with the ERK1/2 inhibitor PD98059 exhibited significantly reduced TNF-α secretion upon LPS challenge (**Figure 9A**), with this impairment associated with decreased TNF-α gene transcription (**Figure 9A**).

To investigate whether exposure to heme influenced LPS-induced activation of ERK1/2, we analysed the phosphorylation status of this MAPK in LPS stimulated THP-1 cells pre-treated with heme. As shown in **Figure 9B**, THP-1 monocytes pre-exposed to heme exhibited significantly impaired activation of ERK1/2 following LPS challenge.

### Monocytes pre-treated with heme exhibit impaired glycolytic responses upon LPS challenge

**Figure 10:**
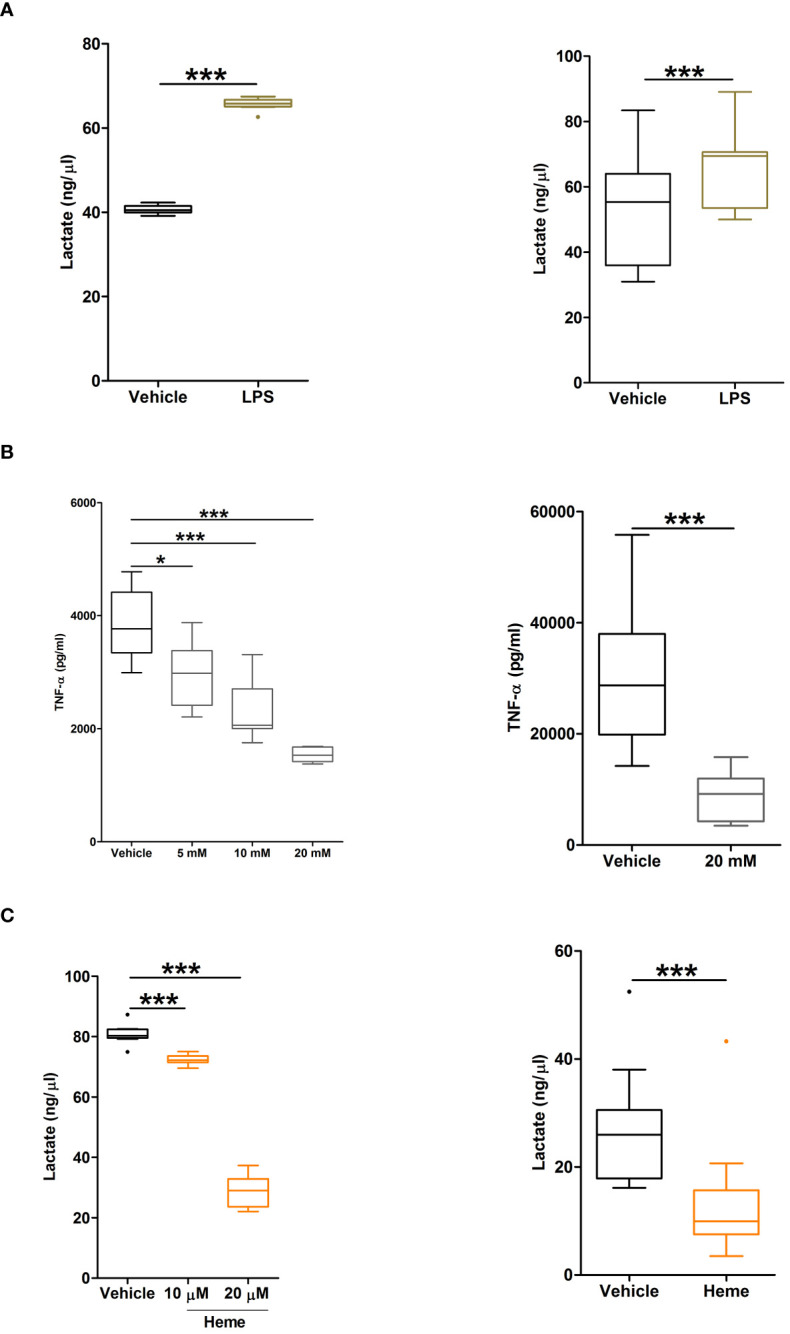
Heme pre-treatment modulates lipopolysaccharide (LPS)-induced metabolic reprogramming in human monocytes. **(A, B)** Concentrations of lactate measured in supernatants of THP-1 cells (left panel, n=9) and PBMCs (right panel, n=11) challenged with 1 µg/ml LPS or vehicle control for 4 hours. **(B)** Concentration of tumour necrosis factor-alpha (TNF-α) detected in supernatants of lipopolysaccharide (LPS) challenged THP-1 cells (left panel) or PBMCs (right panel, n=11) pre-treated for 1 hour with 5–20 mM 2DG. For THP-1 cells, data represents n=21 (vehicle, 5 mM and 10 mM) and n=10 (20 mM). **(C)** Lactate concentrations in supernatants of LPS stimulated THP-1 cells (left panel, n=10) and PBMCs (right panel, n=11) pre-treated for 4 hours with 10–20 µM heme or vehicle control. *p<0.05, ***p<0.0005.

Confirming that metabolic reprogramming towards increased glycolysis precedes TNF-α production by LPS stimulated monocytes (34), we detected significantly higher concentrations of lactate in supernatants of LPS challenged THP-1 cells and PBMCs when compared to vehicle treated controls (**Figure 10A**). Moreover, inhibition of glycolysis, via pre-treatment with the glucose analogue 2-DG, significantly reduced TNF-α production by THP-1 monocytes and PBMCs (**Figure 10B**).

To determine the effect of heme exposure on the glycolytic response of monocytes, we measured lactate concentrations in supernatants of LPS stimulated THP-1 monocytes and PBMCs that had been pre-treated with heme. As shown in **Figure 10C**, prior exposure to heme significantly reduced lactate production by both THP-1 monocytes and PBMCs following LPS challenge.

## Discussion

Evident in the minutes, hours and days following injury, systemic immune suppression is a well-documented consequence of major trauma and severe burns (2, 30, 35, 36), yet the mechanisms underlying its development are poorly understood. Here, through combining *in vitro* assays with *ex vivo* analyses of blood samples acquired from over 200 thermal and traumatically-injured patients during the ultra-early (≤1 hour) and acute (4–72 hours) injury phase, we have provided evidence that suggests a post-injury elevation in circulating concentrations of heme may contribute to the onset of endotoxin tolerance, a phenomenon that predisposes critically-ill patients to the development of HAIs and sepsis (6).

To the best of our knowledge, we are the first human-based study to report that severe thermal injury results in impaired pro-inflammatory cytokine production by LPS challenged whole blood leukocytes. Our direct demonstration of post-burn endotoxin tolerance builds upon observations described in a previous prospective study of severe burns patients that measured the expression of HLA-DR on circulating monocytes (37). An antigen, whose surface levels positively correlate with LPS-induced TNF-α production (38), HLA-DR expression was shown to be reduced over time post-burn, with levels remaining persistently low in patients who subsequently developed septic shock (37). Highlighting the rapidity by which systemic immune suppression develops post-burn, our *ex vivo* analyses of leukocyte function was performed on blood samples acquired within a median time of 17 hours post-injury. When combined with our previous observations of impaired neutrophil function on day 1 post-burn (35), our work collectively demonstrates that the CARS response is initiated within hours of severe thermal injury.

Suggesting a relationship between tissue damage and systemic immune tolerance, pre-clinical models of liver crush injury and trauma haemorrhage have linked post-injury elevations in plasma concentrations of heme to impaired innate immune responses and an increased susceptibility to bacterial infection (17, 18). Compared to healthy controls, we found trauma patients presented ≤1 and 4–12 hours post-injury with significantly higher plasma concentrations of total heme, an observation that supports the findings of a previous study that detected elevated concentrations of heme in blood samples obtained from six trauma patients within 24 hours of injury (17). For the first time, we have also shown that within 24 hours of injury, circulating concentrations of heme are significantly elevated in severe burns patients, with levels returning to those measured in healthy controls by day 3 post-burn. On day 1 of injury, heme concentrations were positively associated with the frequency and absolute number of FRC, suggesting that haemoglobin released from damaged red blood cells is a source of circulating heme in the immediate post-burn phase. Indeed, previous studies have demonstrated direct red blood cell damage, spherocytosis and FRC release in severely injured burns patients (39–41). In contrast, trauma patients presented at all study time-points with FRC counts and frequencies comparable to those recorded in healthy controls. Pointing towards damaged tissue as a potential source of heme post-trauma, a positive correlation was observed between circulating levels of G-actin and heme in blood samples acquired within 1 hour of injury. In addition to trauma-induced hemolysis, results of pre-clinical studies have shown that transfusion of stored red blood cells can increase circulating heme levels (18). However, this medical intervention is unlikely to have influenced the peak concentrations of heme we measured ≤1 hour post-trauma and on day 1 of burn injury given that the number of patients within these cohorts who received blood transfusions prior to study enrolment were 4 and 1 respectively.

A multi-component scavenging system comprised of the plasma residing proteins hemopexin, haptoglobin and albumin is responsible for the clearance of circulating heme (23). Compared to healthy controls, burns and trauma patients presented on days 1 and 3 post-injury with significantly reduced circulating concentrations of hemopexin and albumin. Whilst no study to our knowledge has reported upon how traumatic injury modulates hemopexin levels in humans, our findings in burns patients are in agreement with a previous study that described an early (day 1) and persistent (day 5) reduction in plasma hemopexin concentrations following severe thermal injury (20). As hemopexin exhibits extremely high affinity for heme, a post-injury decline in its circulating levels would not only reduce heme clearance rates but would result in a greater abundance of active free heme. Thus, when combined with the concurrent reduction we detected in albumin levels, it is conceivable that whilst we found no difference in plasma concentrations of total heme between healthy controls and both burns and trauma patients on day 3 post-injury, the proportion of free biologically active heme may have been higher. As reference, the only study to our knowledge to have reported upon circulating levels of free heme post-injury recorded concentrations of 10 µM on day 1 of injury (17).

HO-1 is a stress responsive enzyme that catalyses the conversion of intracellular heme into biliverdin, free iron and carbon monoxide (24). In line with the observations of Rittirsch and colleagues, who detected increased HO-1 gene expression in leukocytes isolated from severe trauma patients (25), we measured, relative to healthy controls, significantly higher HO-1 mRNA levels in PBMCs obtained from major trauma patients 4–12 and 48–72 hours post-injury. In contrast, we found that thermal injury had no effect upon HO-1 gene expression in PBMCs. This latter observation contradicts findings reported in rodent models of thermally-induced injuries, where heat stress was shown to increase HO-1 expression in muscle tissue and hepatocytes (42, 43), and results of a human-based study that revealed increased HO-1 protein expression in skin samples acquired from burns patients on day 5 post-injury (22). Differences in experimental design (rodents Vs humans), the cell types studied (leukocytes, hepatocytes, skin and muscle tissue), severity of injury and sample timings (hours Vs days) could explain these discordant findings. Mechanistically, we speculate that our observation of a trauma but not thermal injury-induced increase in HO-1 expression in PBMCs could be due, in part, to differences in the circulating inflammatory milieu. For example, whilst we found that levels of IL-10, a known inducer of HO-1 expression in leukocytes (44, 45), were not increased in our cohort of burns patients at days 1 or 3 post-injury, we have previously shown that concentrations of this cytokine are elevated <1 and 48–72 hours following major trauma (2). Aligning with findings reported in a murine model of hindlimb ischemia, where increased HO-1 expression promoted activation of the NLRP3 inflammasome (46), Li et al. recently demonstrated that a heat shock-induced up-regulation in HO-1 triggered macrophage ferroptosis and IL-1β-mediated inflammation (47). With high HO-1 expression linked to vascular and cellular injury, as well as exacerbated inflammatory responses (46, 47), *could the increased HO-1 expression that has been detected in skin samples of burns patients negatively impact upon the process of wound healing* (22)? This question is worthy of investigation given that administration of ferroptosis inhibitors has been reported, across multiple rodent models, to accelerate wound repair (48). Interestingly, changes in ferroptosis-related genes (FRGs) were recently identified in leukocytes isolated from thermally-injured patients, with these changes associated with survival (49). Given that we detected increased HO-1 expression in PBMCs of trauma patients, and HO-1 is implicated in ferroptosis (47), future studies should investigate whether the expression of FRGs are also altered post-trauma and, if so, how this impacts upon clinical outcomes. Based on existing literature, it is currently unclear as to what the potential immunological consequences of a trauma-induced increase in HO-1 expression would be. For instance, whilst some studies have shown that exposure to heme-derived carbon monoxide inhibits LPS-induced pro-inflammatory cytokine production by macrophages, and enhances their expression of IL-10 (50, 51), others have reported that by promoting activation of the NLRP3 inflammasome, carbon monoxide enhances the bacterial clearance capacity of macrophages (52). Further studies are therefore needed in order to establish how post-injury increases in HO-1 may impact upon the hosts response to pathogenic challenge.

Via its ability to: (i) inhibit the phagocytic activity of neutrophils and alveolar macrophages (18, 53), (ii) suppress neutrophil chemotaxis (53) and (iii) reduce the expression of TLRs on the surface of circulating neutrophils (17), heme has been assigned an immune suppressive DAMP. Based on the data presented in this manuscript, we suggest that the induction of endotoxin tolerance is an additional immune suppressive property of heme. Supported by our *ex vivo* analyses, which revealed a negative association between plasma heme concentrations and LPS-induced TNF-α and IL-6 production by whole blood leukocytes on day 1 of burn injury, we found that pre-treating THP-1 cells and primary human monocytes with heme significantly reduced their production of TNF-α upon subsequent LPS stimulation. Thus, as suggested for other endogenous ligands of TLR4 such as HSP-70, HMGB-1 and calprotectin (1, 9–12, 54), we propose that the immediate release of heme from spherocytes and damaged tissues contributes to the development of endotoxin tolerance in monocytes. Interestingly, we have previously shown, at our <1 and 48–72 hour post-injury sampling time-points, that trauma results in a significant elevation in both the percentage of TLR4^+^ monocytes and TLR4 surface density (2). We speculate that this rapid and prolonged up-regulation in the expression of the heme receptor may contribute to the ultra-early and persistent induction of endotoxin tolerance by increasing the sensitivity of circulating monocytes to the immune suppressive actions of this DAMP. This proposed mechanism may also apply to burns patients given that increased expression of TLR4 has been recorded for monocytes isolated from thermally-injured subjects on day 1 post-burn (55).

Alongside its immune suppressive actions, heme is a potent activator of innate immune responses, with studies demonstrating that exposure to this DAMP induces cytokine production, extracellular trap formation and ROS generation by neutrophils (14–16, 56), and stimulates the secretion of TNF-α by macrophages (13). Via its immune activatory and suppressive properties, the ultra-early (<1 hour) and acute (4–24 hours) elevation in plasma heme concentrations that occurs following severe burns and trauma therefore offers a potential mechanistic explanation for how SIRS and CARS responses can be induced simultaneously following major injury (2, 57). Although not proving causality, our data showing circulating heme concentrations were positively associated with plasma levels of pro and anti-inflammatory cytokines on days 1 and 3 post-thermal and traumatic injury lend support to the idea that heme may promote and/or prolong the state of systemic immune dysregulation that occurs following major injury. Conceptually, it is conceivable that exposure to heme triggers immediate functional responses by immune cells that results in a state of functional exhaustion and thus impaired anti-microbial responses to secondary stimulation.

Our investigations into the mechanisms underlying heme-induced endotoxin tolerance revealed pre-treatment with this DAMP resulted in impaired LPS-induced activation of ERK1/2 and NF-κB as well as altered metabolic reprogramming, with heme treated monocytes exhibiting reduced lactate production upon endotoxin challenge. Previous studies in the setting of DAMP-induced immune suppression have revealed that endotoxin tolerance triggered by mtDNA pre-treatment is also associated with reduced activation of NF-κB following LPS stimulation (58), whilst neutrophils pre-exposed to whole mtDAMP preparations were found to generate significantly lower amounts of lactate upon secondary challenge (30). Although not investigated in the context of burn injury, reduced glycolytic responses have been reported in endotoxin tolerant monocytes isolated from critically-ill septic patients who themselves present with reduced circulating concentrations of hemopexin (59, 60). Importantly, both the impaired cytokine producing capacity and glycolytic responses of these tolerant monocytes could be reversed by treating patients with recombinant interferon gamma (59). Thus, correcting DAMP-induced suppression of monocyte metabolic reprogramming could represent a novel means of restoring immune function post-burn.

Adding to a growing body of literature that suggests elevated circulating levels of DAMPs are associated with poor clinical outcomes following major injury (1, 61–63), we found that participants presenting with significantly higher plasma concentrations of heme on day 1 of burn injury had higher odds of mortality. Probing this relationship further, we established that, independent of patient age, gender and % TBSA, a difference of 6.5 µM in the circulating concentration of total heme corresponded to a relative increase in the odds of mortality of 52%. Furthermore, on day 1 of burn injury, total heme levels could moderately discriminate between survivors and non-survivors, with prognostic modelling generating an AUROC value of 0.78. Studies that have shown exposure to heme induces oxidative injury, promotes endothelial dysfunction and potentiates platelet activation and aggregation offer potential mechanistic explanations for this relationship between post-burn elevations in heme and poor clinical outcomes (27).

Given the potential clinical relevance of elevated heme levels, *could therapeutic approaches aimed at reducing circulating heme concentrations improve the outcomes of severely-injured patients?* One potential strategy could be to restore, via replenishment therapy, plasma concentrations of hemopexin, the potent heme scavenger whose circulating levels we found were significantly reduced in the hours and days following thermal and traumatic injury (64). Providing support to this idea, Larsen et al, in a model of severe sepsis, found that administration of hemopexin to mice with elevated plasma levels of free heme reduced mortality rates by 22% (65). Similar findings were also reported in a murine model of trauma haemorrhage and bacterial infection, where hemopexin treatment improved post-pneumonia survival rates (18). Given that this reduction in mortality was accompanied by a significant decrease in bacterial load within the lung (18), hemopexin administration could potentially be a means of preventing heme-induced immune suppression. In line with this narrative, we found that culturing monocytes in 20% FCS prevented heme-induced suppression of TNF-α production upon LPS stimulation. Furthermore, it was recently demonstrated *in vitro* that hemopexin supplementation of plasma samples obtained from patients experiencing significant intravascular hemolysis could counteract heme driven suppression of macrophage phagocytic activity (66). We were unable to perform similar experiments to address whether heme scavenging could prevent the induction of endotoxin tolerance by plasma samples obtained burns and trauma patients due to the small volumes of blood collected at our study time-points.

Conducted at a single major trauma and burns centre, our findings require validation in larger independent cohorts. Indeed, as our study was not designed to test whether post-injury changes in plasma heme levels influenced patient outcomes, the analyses we conducted are exploratory rather than confirmatory. The association we report between elevated heme concentrations on day 1 of burn injury and an increased risk of mortality may be limited to our dataset, meaning that it cannot be generalised to all thermally-injured patients from whom blood samples could be obtained within 24 hours of injury. Rather than a definitive finding, this observation should therefore serve as a hypothesis for future adequately powered prospective studies to test.

In summary, we have shown that major traumatic and severe thermal injury results in an immediate elevation in plasma concentrations of total heme and provided evidence that suggests raised heme levels post-burn contribute to the development of endotoxin tolerance. By demonstrating that the post-injury impairment in LPS-induced pro-inflammatory cytokine production by whole blood leukocytes can be recapitulated *in vitro* by pre-treating monocytes from healthy volunteers with physiological doses of heme, our data provides further support to the concept of post-injury DAMP-induced immune suppression (7). Interestingly, heme has recently been identified as an inducer of trained immunity, where depending upon the context and timing of secondary insult, it was shown to elicit either protective or deleterious effects on host immune responses (67). Proposed to result from maladaptive myelopoiesis, it was suggested that the deleterious effects of heme mediated trained immunity could manifest as impaired immune responses to infection (67). On this note, it is of interest that monocytes with reduced HLA-DR expression, a phenotype associated with impaired LPS-triggered TNF-α production (38), have been detected in the circulation of non-survivors of thermal injury at day 28 post-burn (68). Thus, future studies that examine the long-term myeloid adaptations that occur following thermal injury, and their relationship with heme, could offer mechanistic insights into the long-term immune dysregulation experienced by this patient group. Finally, as both traumatic and thermal injury resulted in the depletion of plasma proteins involved in heme scavenging, our data raises the possibility that, by counteracting heme-induced immune suppression, replenishment therapy with the heme scavenger hemopexin could improve the clinical outcomes of critically-ill patients.

## Data availability statement

The raw data supporting the conclusions of this article will be made available by the authors, without undue reservation.

## Ethics statement

The studies involving humans were approved by the North Wales Research Ethics Committee - West (REC reference:13/WA/0399, Protocol Number : RG_13-164) for the BBATS study and the West Midlands, Coventry and Warwickshire Research Ethics Committee (REC reference:16/WM/0217) for the SIFTI-2 study (trial registration number:NCT04693442). Healthy controls were recruited in accordance with the ethical approval granted by the University of Birmingham Research Ethics Committee (Ref: ERN_12-1184). The studies were conducted in accordance with the local legislation and institutional requirements. The participants provided their written informed consent to participate in this study.

## Author contributions

ST: Data curation, Formal analysis, Investigation, Writing – review & editing. TN: Data curation, Formal analysis, Investigation, Writing – review & editing. JB: Formal analysis, Methodology, Writing – review & editing. KM: Data curation, Investigation, Writing – review & editing. AA: Data curation, Investigation, Writing – review & editing. JS: Data curation, Investigation, Writing – review & editing. Y-YC: Data curation, Investigation, Writing – review & editing. AS: Data curation, Investigation, Writing – review & editing. AB: Conceptualization, Funding acquisition, Writing – review & editing. PH: Investigation, Supervision, Writing – review & editing. NM: Funding acquisition, Writing – review & editing, Investigation. JL: Investigation, Supervision, Writing – review & editing, Funding acquisition. JH: Funding acquisition, Investigation, Supervision, Conceptualization, Data curation, Formal analysis, Writing – original draft.

## Supplementary material

Supplementary Figure 1 Circulating endotoxin concentrations post-burn. Comparison of endotoxin levels in serum samples obtained from 5 severe burns patients on day 1 of injury and 5 healthy controls (HC). **p<0.005.

Supplementary Figure 2 Prospective analysis of circulating hemopexin and albumin concentrations following severe thermal injury. Longitudinal assessment of plasma hemopexin (left panel, n=73) and serum albumin (right panel, n=67) concentrations in severe burns patients across days 1 and 3 post-injury. **p<0.005, ***p<0.0005.

Supplementary Figure 3 Heme treatment does not activate or induce cytotoxicity in THP-1 cells. **(A)** Concentration of tumour necrosis factor-alpha (TNF-α) detected in supernatants collected from cultures of THP-1 cells treated for 4 hours with 20 µM heme (n=15) or 1 µg/ml lipopolysaccharide (LPS, n=8). ***p<0.0005 Vs. vehicle. ^##^p<0.005. **(B)** Lactate dehydrogenase (LDH) activity measured in supernatants acquired from cultures of THP-1 cells treated for 4 hours with vehicle (veh) control, 10–20 µM heme or 10 µM staurosporine (ST, n=6). ***p<0.0005 Vs. untreated (unt).
