# Supplementary figures and images for: Severe thermal and major traumatic injury results in elevated plasma concentrations of total heme that are associated with poor clinical outcomes and systemic immune suppression
Source: Front Immunol. 2024 Jun 14;15:1416820. doi: 10.3389/fimmu.2024.1416820 (PMC11211257; doi:10.3389/fimmu.2024.1416820)

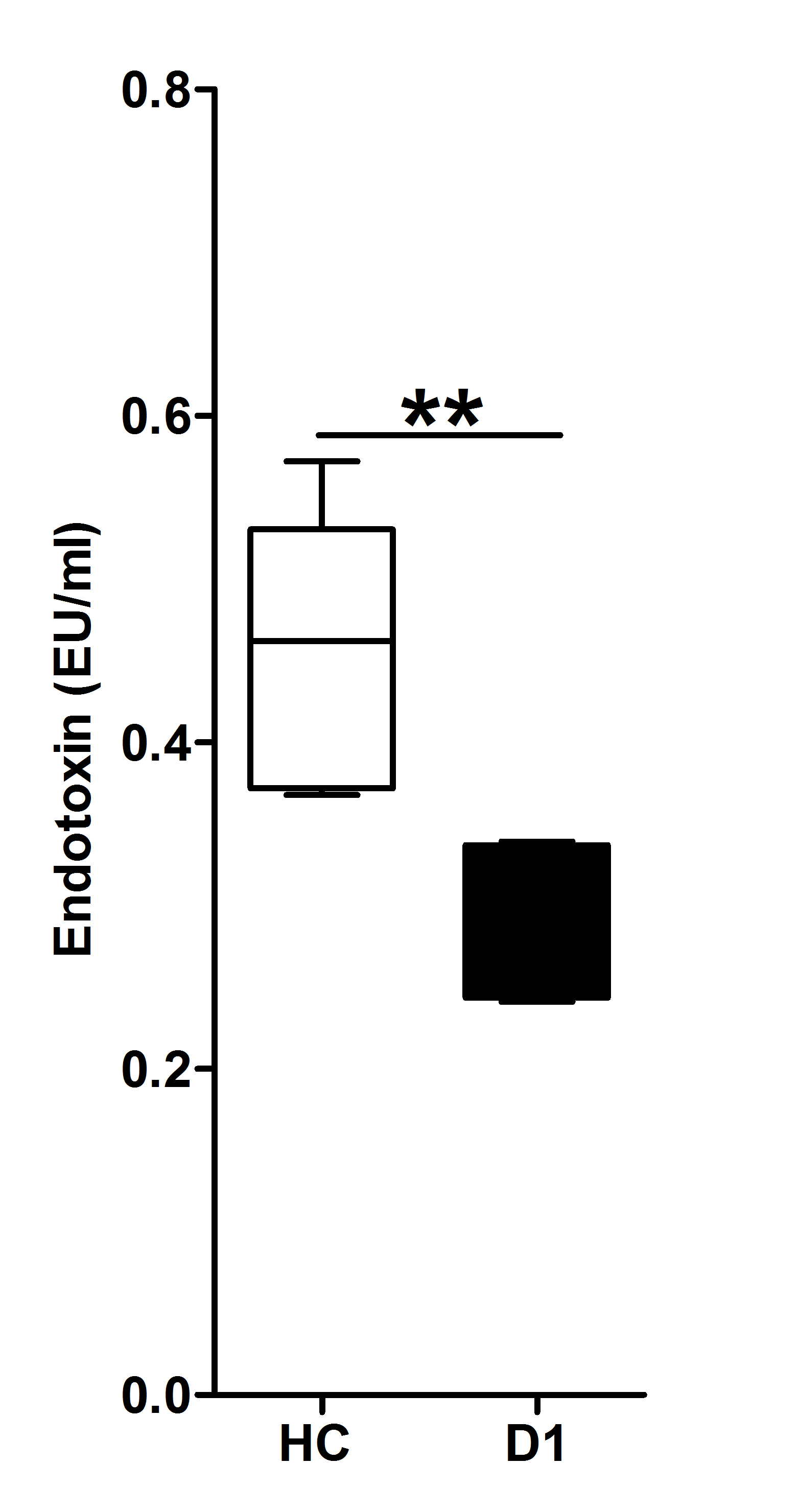

Supplement: Supplementary Figure 1 — Circulating endotoxin concentrations post-burn. Comparison of endotoxin levels in serum samples obtained from 5 severe burns patients on day 1 of injury and 5 healthy controls (HC). **p<0.005. [file Image_1.jpeg]

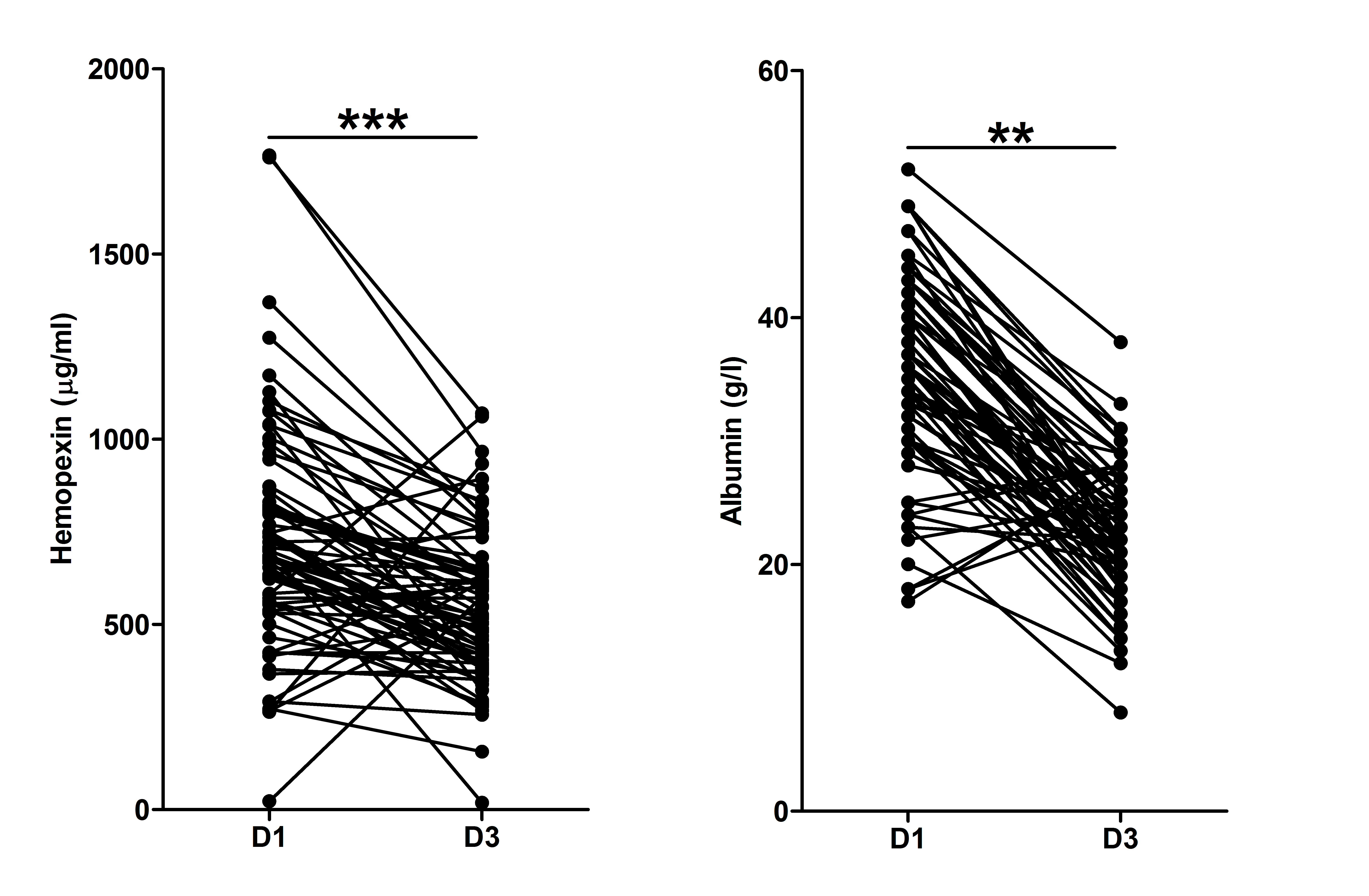

Supplement: Supplementary Figure 2 — Prospective analysis of circulating hemopexin and albumin concentrations following severe thermal injury. Longitudinal assessment of plasma hemopexin (left panel, n=73) and serum albumin (right panel, n=67) concentrations in severe burns patients across days 1 and 3 post-injury. **p<0.005, ***p<0.0005. [file Image_2.jpeg]

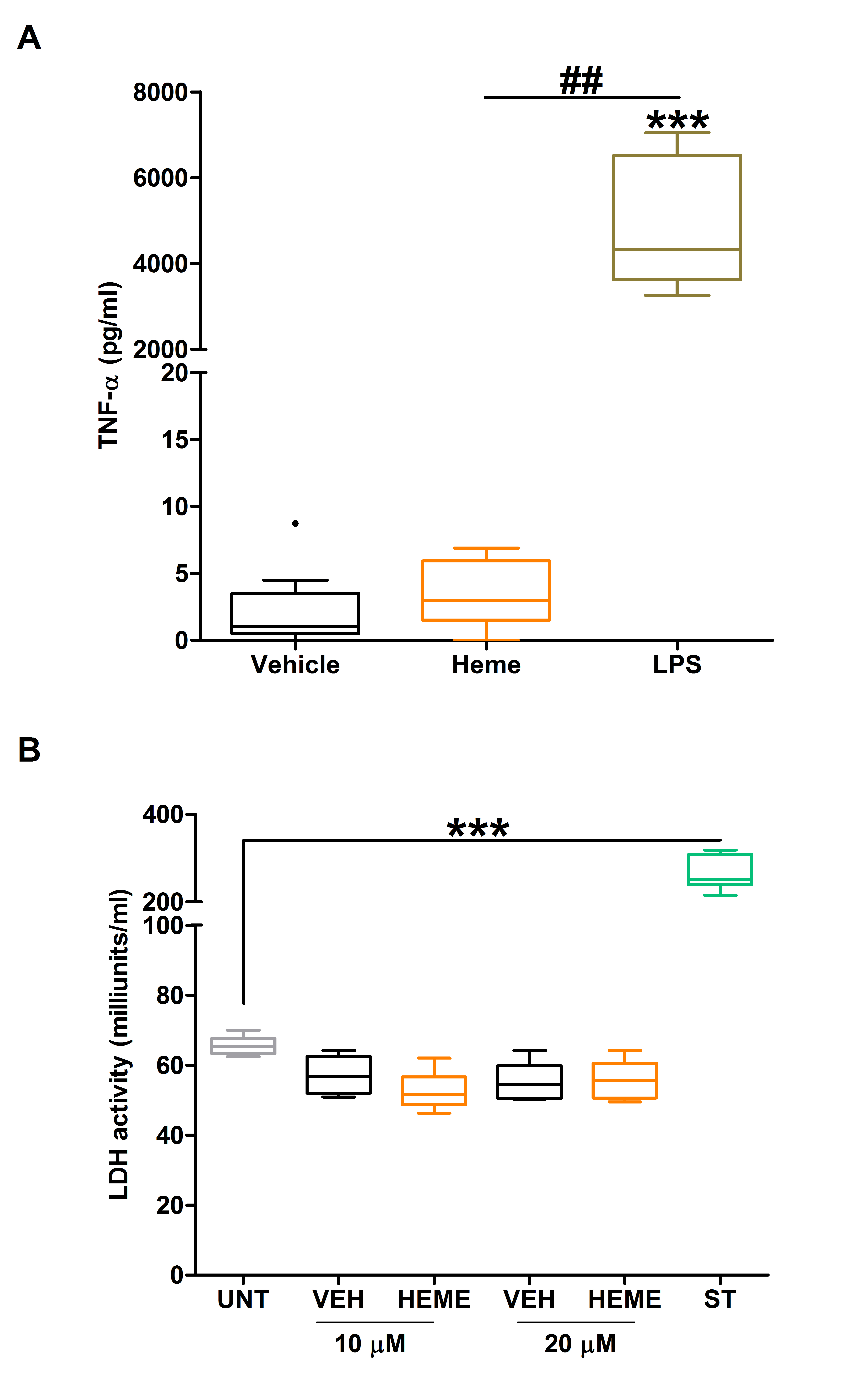

Supplement: Supplementary Figure 3 — Heme treatment does not activate or induce cytotoxicity in THP-1 cells. (A) Concentration of tumour necrosis factor-alpha (TNF-α) detected in supernatants collected from cultures of THP-1 cells treated for 4 hours with 20 µM heme (n=15) or 1 µg/ml lipopolysaccharide (LPS, n=8). ***p<0.0005 Vs. vehicle. ##p<0.005. (B) Lactate dehydrogenase (LDH) activity measured in supernatants acquired from cultures of THP-1 cells treated for 4 hours with vehicle (veh) control, 10–20 µM heme or 10 µM staurosporine (ST, n=6). ***p<0.0005 Vs. untreated (unt). [file Image_3.jpeg]
