# Supplementary material for: Severe thermal and major traumatic injury results in elevated plasma concentrations of total heme that are associated with poor clinical outcomes and systemic immune suppression
Source: Front Immunol. 2024 Jun 14;15:1416820. doi: 10.3389/fimmu.2024.1416820 (PMC11211257; doi:10.3389/fimmu.2024.1416820)
Supplement: Supplementary file 4 [file Table_1.docx]

**Supplementary Table 1. Correlative analyses examining the relationship between patient age and plasma concentrations of heme in thermal and traumatically-injured patients.**

|  | **Day 1** | **Day 3** | **<1H** | **4-12H** | **48-72H** |
| --- | --- | --- | --- | --- | --- |
| **Burns** | R= 0.08  (-0.138-0.290)  p= 0.462  n= 88 | R= 0.116  (-0.108-0.330)  p= 0.295  n= 83 | N/A | N/A | N/A |
| **Trauma** | N/A | N/A | R= 0.112  (-0.086-0.302)  p= 0.254  n= 106 | **R=0.238**  **(0.050-0.410)**  **p= 0.011**  **n= 113** | R=-0.110  (-0.303-0.092)  p= 0.272  n= 102 |

95% confidence intervals are presented in paratheses.

Significant associations according to Spearman’s rank correlation coefficient are indicated in bold font.
