# Supplementary material for: Severe thermal and major traumatic injury results in elevated plasma concentrations of total heme that are associated with poor clinical outcomes and systemic immune suppression
Source: Front Immunol. 2024 Jun 14;15:1416820. doi: 10.3389/fimmu.2024.1416820 (PMC11211257; doi:10.3389/fimmu.2024.1416820)
Supplement: Supplementary file 5 [file Table_2.docx]

**Supplementary Table 2. Demographic and clinical data of burns patients categorised according to**

**survival and sepsis status.**

| **Characteristic** |  | | | | | |
| --- | --- | --- | --- | --- | --- | --- |
|  | **Survival Status** | | | **Sepsis Status** | | |
|  | **Survivors**  **(n=74)** | **Non-survivors**  **(n=14)** | **P value** | **Septic**  **(n=42)** | **Non-septic**  **(n=37)** | **P value** |
| Age, years (range)  Gender,  *Male*  *Female*  % TBSA (range)  % FT TBSA (range)  Inhalation injury  *Yes*  *No*  Mechanism of injury  *Flash, n (%)*  *Flame, n (%)*  *Flame and flash, n (%)*  *Electrical, n (%)*  *Scald, n (%)*  ABSI (range)  Baux (range)  rBaux (range)  Day 1 SOFA (range)  Day 1 Denver (range)  ICU free days (range)  Hospital free days (range) | 44 (16-83)  59  15  34 (15-80)  17 (0-75)  32  42  6 (8)  60 (82)  4 (5)  1 (1)  3 (4)  7 (2-13)  77 (34-130)  85 (39-147)  6 (0-17)  2 (0-7)  18 (0-30)  5 (0-24) | 55 (22-76)  9  5  45 (15-85)  30 (0-71)  7  7  0 (-)  13 (93)  1 (7)  0 (-)  0 (-)  10 (5-14)  99 (72-132)  108 (72-149)  10 (4-15)  4 (1-7)  4 (0-29)  0 | **0.014**  n.s  0.06  **0.02**  n.s  n.s  n.s  n.s  n.s  n.s  **0.004**  **0.004**  **0.01**  **0.001**  **0.001**  **<0.0001**  **0.005** | 46 (16-83)  31  11  43 (15-80)  28 (0-75)  28  14  1 (2)  39 (94)  1 (2)  1 (2)  0 (-)  8 (2-13)  89 (34-130)  100 (50-147)  9 (1-17)  3 (0-7)  9 (0-30)  1 (0-11) | 45 (19-79)  30  7  25 (15-60)  7 (0-58)  6  31  4 (11)  26 (70)  4 (11)  0 (-)  3 (8)  6 (2-10)  70 (39-104)  72 (39-105)  4 (0-13)  1 (0-4)  24 (0-30)  7 (0-20) | n.s  n.s  **<0.0001**  **<0.0001**  **<0.001**  n.s  **<0.05**  n.s  n.s  n.s  **0.004**  **0.001**  **<0.0001**  **<0.0001**  **<0.0001**  **<0.0001**  **<0.0001** |

ABSI, Abbreviated Burn Severity Index; SOFA, Sequential Organ Failure Assessment; TBSA, Total body surface area burn.
